# The influence of polymer purification on the efficiency of poly(3-hexylthiophene):fullerene organic solar cells

**DOI:** 10.1038/srep23651

**Published:** 2016-03-31

**Authors:** James H. Bannock, Neil D. Treat, Michael Chabinyc, Natalie Stingelin, Martin Heeney, John C. de Mello

**Affiliations:** 1 Centre for Plastic Electronics, Imperial College London, London SW7 2AZ, UK; 2 Department of Chemistry, Imperial College London, London SW7 2AZ, UK; 3 Department of Materials, Imperial College London, London SW7 2AZ, UK; 4 Materials Research Laboratory, University of California Santa Barbara, Santa Barbara, California 93117, USA

## Abstract

We report the influence of different polymer purification procedures on the photovoltaic performance of bulk heterojunction solar cells formed from binary blends of poly(3-hexylthiophene) (P3HT) and fullerenes. Selective Soxhlet extractions and metal scavenging agents were used to remove residual monomer, magnesium salt by-products and catalyst from high-weight P3HT (Mw 121 kg/mol, PDI 1.8, RR 99%) synthesised by the Grignard metathesis (GRIM) polymerization route. Using phenyl-C61-butyric acid methyl ester (PC_60_BM) as an electron acceptor, we observed an increase in average power conversion efficiency from 2.3 to 4.8% in going from crude to fully purified material. Using indene-C_60_ bisadduct (IC_60_BA) in place of PC_60_BM, we observed a further increase to an average value of 6.6% - high for a bulk heterojunction formed from a binary blend of P3HT and C_60_ fullerene derivatives.

Semiconducting polymers offer the prospect of solution processable electronic devices that can be deposited over large areas at minimal cost^1^,^2^. For the technology to succeed, however, significant improvements in the performance, cost and reliability of the constituent functional materials are required. Much research has focused on developing new classes of semiconducting polymers with improved charge transport and/or light-harvesting properties, which has led for instance to organic photovoltaic (OPV) devices with power conversion efficiencies in excess of 10%^3^,^4^,^5^. However, owing to the complexity of their syntheses, few of the materials so far reported are capable of reaching viable price points for commercial application^6^. Furthermore, the frequent use of stoichiometric quantities of organotin reagents (during monomer preparation and subsequent polymerization) presents considerable safety challenges, which further add to the cost of the end material^7^. Even setting aside cost considerations, sourcing high performance semiconducting polymers in industrially useful quantities is a considerable challenge, with batch-to-batch variability (in terms of molecular weight distributions, structural order and purity) leading to inconsistent processing properties and device efficiencies^8^. New synthesis routes and purification procedures that can provide consistent material, batch after batch, are urgently required if semiconducting polymers are to reach commercial maturity.

One of the very few materials that can be produced cost-effectively in large quantities is regioregular poly(3-hexylthiophene) (P3HT), an extensively studied derivative of polythiophene, first developed in the early 1990’s to enable solution processing of the conjugated thiophene chain. Of the various synthesis procedures reported, Loewe and McCullough’s Grignard metathesis (GRIM) polymerization method is arguably the simplest, involving a two-stage, single-pot process^9^,^10^. Requiring a single dibrominated thiophene monomer and off-the-shelf reagents and catalysts, it provides a more cost-effective route to semiconducting polymers than other commonly used coupling routes, e.g. Stille, Suzuki and direct arylation^11^,^12^. Beyond thiophene derivatives, the GRIM method has been successfully applied to a broad range of other semiconducting polymers, including polyfluorenes and poly-*p*-phenylenes^13^. There is therefore considerable interest in developing treatment protocols that can extract the optimum performance from GRIM-synthesised materials.

Here we look specifically at the influence of purification protocols on the photovoltaic performance of bulk heterojunction solar cells formed from binary blends of GRIM-synthesised P3HT and fullerenes. In this work we employ P3HT of weight-average molecular weight (Mw) ~120 kg/mol and regioregularity (RR) >99% – high Mw, high RR P3HT having previously been shown to yield high efficiencies in bulk heterojunction solar cells^14^.

## Results and Discussion

**Figure 1:**
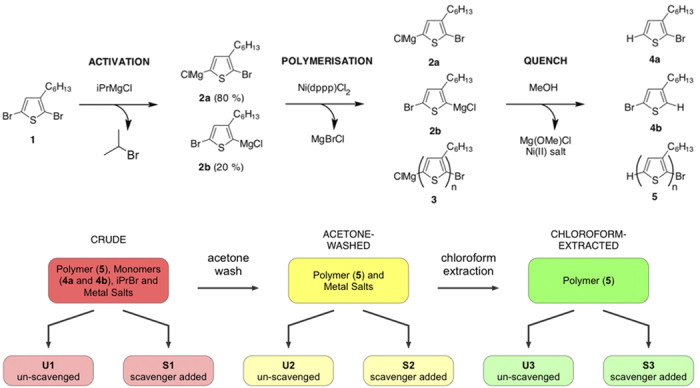
Synthesis of poly(3-hexylthiophene) by Grignard metathesis (GRIM) polymerization (top) and flowchart describing the purification steps used to obtain samples U1-3 and S1-3 (bottom).

Figure 1 (top) describes the two-step procedure for synthesising P3HT by Grignard metathesis polymerization, followed by a quench in methanol to terminate the reaction. In the first step 2,5-dibromo-3-hexylthiophene (**1**) is activated with one mole equivalence of isopropylmagnesium chloride (iPrMgCl) in anhydrous tetrahydrofuran (THF), producing an approximate 80:20 mix of thienyl-Grignard isomers (**2a** and **2b**, respectively). A catalytic amount of nickel(II)[1,3-bis(diphenylphosphino)propane] chloride, Ni(dppp)Cl_2_, is then added, initiating polymerization of the dominant **2a** intermediate^10^,^15^. After polymerization has proceeded to the desired extent, excess methanol is added to the reaction mixture to de-activate the remaining Grignard-moieties, i.e. the unpolymerized monomers (**2a** and **2b**) and the polymer **3**, forming **4a**, **4b** and **5** respectively.

By-products are formed at each stage of the GRIM procedure. One mole equivalence of 2-bromopropane is produced during the activation step. During the polymerization step, the catalyst cross-couples monomer units to the end of the growing polymer chain, producing MgBrCl with each iteration. Upon quenching, Mg(OMe)Cl is produced by reaction of the protic solvent with the Grignard-terminated moieties **2a**, **2b** and **3**. In addition to these by-products, the nickel catalyst remains in the final product mixture. Therefore, prior to purification, at least six separate contaminants may be present in a sample alongside the polymer: 2-bromopropane, MgBrCl, Mg(OMe)Cl, **4a**, **4b** and Ni(II) salt. To highlight the scale of contamination: assuming full conversion of the active monomer species, **4a**, for every 1 g of P3HT obtained there is an expected 2.28 g of impurities, equating to 69.5% impurities by mass in a crude unpurified sample (see Section B of SI for further details).

In the absence of a solvent, the metal salts and polymer are solids, while the remnant monomers and 2-bromopropane are liquids. On the lab scale, removal of these impurities is typically achieved using Soxhlet extraction, which is a common method for extracting/separating mixed chemical species. Soxhlet extraction depends on the ability of a chosen solvent to selectively dissolve and extract specific components through a porous thimble (usually cellulose), leaving any insoluble components behind in the thimble. For materials synthesised via the GRIM route, it is common to first purify P3HT by Soxhlet extraction with methanol to remove the metal salts and monomers, and then extract the polymer from any remaining trapped metal salts by repeating the process with chloroform (which dissolves the polymer but not the metal salts). Additional solvents such as acetone, hexane, heptane or dichloromethane are sometimes used between these steps to remove lower molecular weight chains from the bulk polymer sample (and thereby narrow the molecular weight distribution)^16^,^17^,^18^.

For the current work, a non-standard purification procedure was employed using acetone in place of methanol for the first Soxhlet stage. Magnesium salts are sparingly soluble in acetone, meaning Soxhlet extraction with acetone predominantly removes the liquid components (**4a**, **4b** and 2-bromopropane) and very short chain oligomers. The polymer was then extracted from the remnant magnesium salts in the second stage using chloroform as the extracting solvent. (With a relative permittivity of 4.8 and a dipole moment of ~1.1 D, chloroform is insufficiently polar to dissolve the strongly ionic magnesium salts^19^). Using this combination of acetone purification followed by chloroform extraction, it is possible to decouple the influence of monomeric/oligomeric species and magnesium salts on electronic device performance.

Residual catalysts (and other impurities^20^,^21^) may also have a strong influence on electronic device performance^22^,^23^,^24^. To determine the effect of the nickel catalyst on device performance, approximately half of the dry polymer obtained at each stage of purification was re-dissolved in hot solvent – either a mixture of THF and chloroform for the crude sample (which would not fully dissolve in THF alone) or pure THF for the acetone-washed and chloroform-extracted samples. An excess of silica-supported nickel scavenging agent was then added to each solution. After stirring for 15 minutes, the scavenging agent was removed from each solution by vacuum filtration and the filtrate was dried overnight in a vacuum oven, leaving behind a scavenged sample of the polymer (see SI, Sections D and E for experimental details).

In total six samples of P3HT were obtained using the above purification procedures (see lower schematic of Fig. 1): an unscavenged sample of the crude product (U1), an unscavenged sample of the product after acetone washing (U2), an unscavenged sample of the product after subsequent chloroform extraction (U3), plus a further three scavenged samples (S1, S2 and S3) derived from the corresponding unscavenged samples.

### Molecular Weight Characterization

Each of the six polymer samples was analysed by refractive-index size-exclusion chromatography (RI-SEC) to determine its molecular weight distribution (see Figures SI2 and SI3 in SI, Section F). Table 1 records the weight-average molecular weight (Mw), peak molecular weight (Mp) and polydispersity index (PDI) for each sample. The two crude samples (U1 and S1) had very different molecular weights distributions, with S1 being significantly lower in weight-average molecular weight and possessing a higher polydispersity. Since S1 derives directly from U1, the observed change is attributable to the process of applying (and subsequently removing) the metal scavenging agent: U1 exhibited low solubility in THF/chloroform and, in the process of filtering out the scavenging agent, a substantial amount of polymer precipitated in the filter and was lost due to the temperature drop during filtration. The chromatograms are consistent with higher molecular weight chains being preferentially lost during filtration in accordance with expectation. After acetone purification, the solubility of the polymer in THF was greatly improved, and filtration could be carried out without significant loss of material. The remaining samples showed similar molecular weight averages and PDIs to U1, implying the two Soxhlet extractions did not significantly affect the molecular weight distribution of the polymer. Normalised absorption spectra of U1-3 did not differ significantly in shape (see SI4 in SI, Section G) – consistent with their identical molecular weight distributions.

### Analysis by ^1^H Nuclear Magnetic Resonance (NMR) Spectroscopy

**Figure 2:**
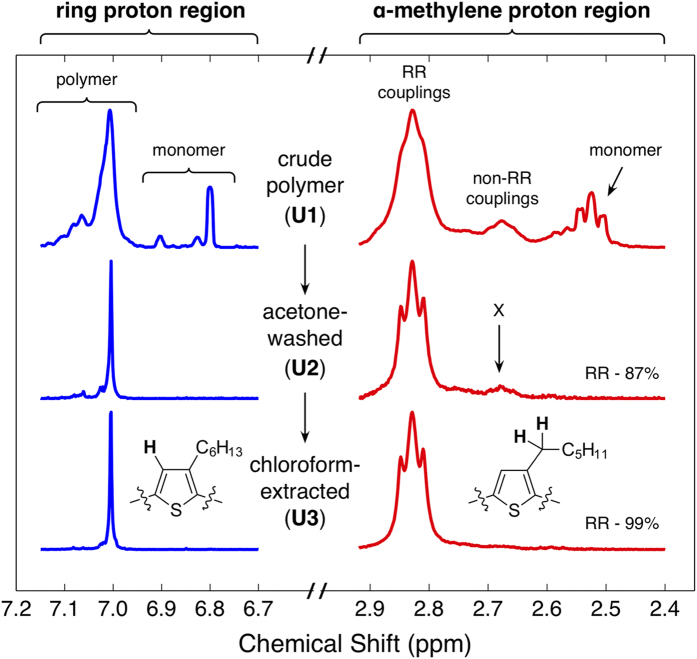
Partial ^1^H NMR specta of un-scavenged samples U1-3 in the ring-proton region (left), and the α-methylene region (right). Inset structures show the corresponding proton environment on the polymer repeat unit.

P3HT has a relatively simple^1^H NMR signature, with two proton environments that are distinct from the deriving monomer, making it straightforward to observe monomer and other hydrocarbon impurities. Spectra were acquired for each of the unscavenged samples (U1-3). Figure 2 shows for each sample the two key regions of the ^1^H NMR spectra, focusing on the ring proton region (left) and the α-methylene proton region (right). Full range ^1^H NMR spectra are provided in Figure SI5 (SI, Section H).

Starting with the most purified material, U3: in the ring proton region there is a single sharp peak at 7.0 ppm that corresponds to the ring proton at the 4-position on the thiophene ring; and in the α-methylene region there is a single structured peak at 2.85 ppm that corresponds to regioregular α-methylene protons on the n-hexyl side-chain.

In contrast, the ^1^H NMR spectrum of the crude polymer U1 exhibits additional signals in both regions of the spectrum, corresponding to the remnant monomers. The additional proton environments at the 2- and 5-positions on the thiophene ring (which are formed after quenching the monomers in methanol) produce signals that overlap with the proton on the polymer repeat unit, causing a broadening of the 7.0 ppm peak. Additional features are also present at 3.87 ppm (see Figure SI5), corresponding to 2-bromopropane.

After purifying the crude sample with acetone (U2), the monomer and 2-bromopropane features are absent from the ^1^H NMR spectrum, indicating their successful removal. The result is a sharp peak at 7.0 ppm along with a well-defined α-methylene peak at 2.85 ppm. In addition to these features, there is a weak signal at 2.6–2.8 ppm (marked X) and a small feature on the de-shielded side of the 7.1 ppm signal, both of which are attributable to non-regioregular (non-RR) couplings^25^.

For asymmetric polythiophene-derivatives, it is standard practice to calculate the regioregularity (RR) from the areas under the RR and non-RR features in the α-methylene region, as defined in Equation (1)


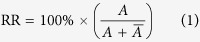


where *A* is the integrated area under the regioregular α-methylene signal and 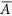 is the integrated area under the non-regioregular α-methylene signal.

The regioregularity of the crude sample (U1) could not be accurately determined because the α-methylene signal of the polymer was obscured by the overlying α-methylene signals of the monomer. The regioregularity of the polymer was determined to be 87% after acetone washing (U2) and 99% after chloroform extraction (U3). Whilst there is no chemical difference between the P3HT in U2 and U3, it was observed that the (salt-containing) U2 sample rapidly gelled in the NMR solvent (CDCl_3_), suggesting its lower apparent regioregularity was a consequence of polymer aggregation.

### Analysis by X-Ray Fluorescence (XRF) Spectroscopy

**Figure 3:**
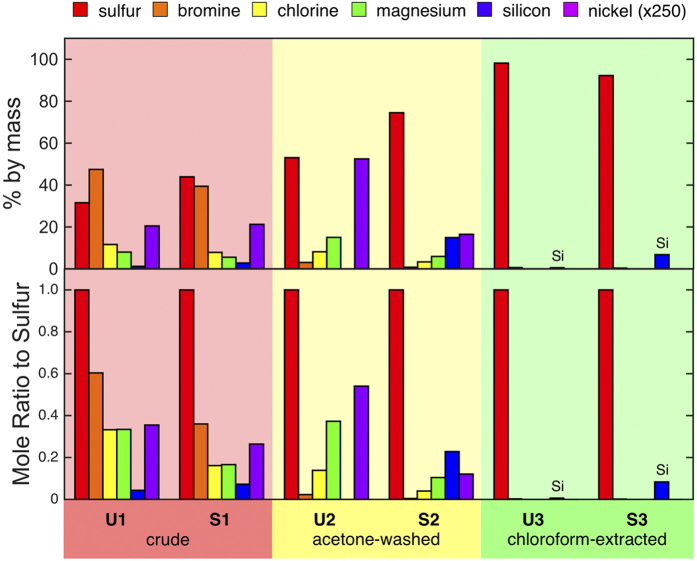
Weight percentage of sulfur, bromine, chlorine, magnesium, silicon and nickel detected in samples U1-3 and S1-3, as determined by X-ray fluorescence spectroscopy (XRF) (top); and molar ratio of each element relative to sulfur (bottom). For samples U1-2 and S1-2 the nickel signal has been scaled by a factor of 250 to make it visible on the Y-scale. Nickel was not detected in chloroform-extracted samples U3 and S3.

All samples were analysed by XRF to quantify their elemental composition. The top panel of Fig. 3 shows in sequence for each of the samples the percentage by mass of sulfur, bromine, chlorine, magnesium, silicon and nickel, while the bottom panel of Fig. 3 shows the molar ratio of each element relative to sulfur (see SI, Section I for methods). The sulfur signal arises from the presence of polymer repeat units and individual monomers.

Analysis of the XRF data for the crude sample (U1) reveals a high concentration of bromine, chlorine and magnesium, indicating the presence of the expected magnesium salt by-products. Magnesium and chlorine are present in approximately equimolar quantities, consistent with the presence of MgBrCl and Mg(OMe)Cl as the principal salt impurities. The higher concentration of bromine relative to Mg and Cl implies that, in addition to MgBrCl, the monomeric species (**4a** and **4b**) and 2-bromopropane are also present, consistent with the ^1^H NMR spectra.

Following acetone purification (U2), the molar ratio of bromine to sulfur was markedly reduced, indicating substantial removal of the monomers (**4a** and **4b**) and 2-bromopropane, which is again consistent with the ^1^H NMR spectra. With the monomer removed, the mass fraction of the polymer in the sample was found to be 91% (assuming that the sulfur originates exclusively from the polymer repeat unit and ignoring bromine end-groups, see SI, Section I). The molar excess of magnesium relative to chlorine implies the presence of unexpected magnesium salts (since the molar ratio of magnesium to chlorine would equal unity if MgBrCl and Mg(OMe)Cl were the only salt impurities). These unidentified salts are likely to arise from metathesis reactions between salts during the monomer quenching. Neither magnesium nor chlorine is detected after chloroform extraction of the polymer (U3), indicating complete removal of the salt by-products, which has the effect of increasing the mass fraction of the polymer in the sample to 99.9%.

Before chloroform extraction, nickel was detected in all samples at <0.01 wt% relative to sulfur, including those that had been treated with the scavenging agent, suggesting the formation of an insoluble nickel salt during synthesis/work-up that prevents at least some of the nickel from coordinating to the scavenger. No nickel was detected after chloroform extraction, indicating that the nickel salts remain behind in the Soxhlet thimble. We note that the metal-scavenged samples, S1 and S2, had significantly lower magnesium contents than the deriving samples U1 and U2. Although the scavenger itself does not coordinate to the magnesium, the filtering step has the effect of (partially) removing insoluble impurities such as magnesium salts, leading to the observed reduction in magnesium concentration. All three scavenged materials (S1, S2 and S3) exhibited an elevated content of silicon, due to contamination by the silica, implying the filtering procedure was not effective at removing the agent.

### PC_60_BM Device Performance

**Figure 4:**
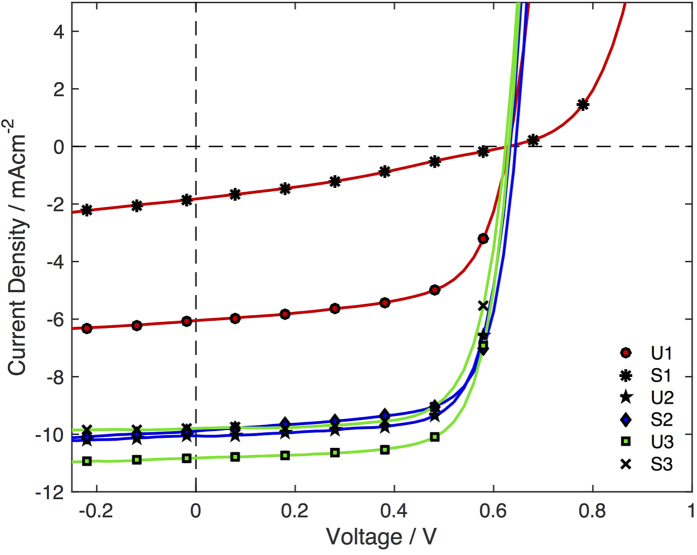
Current density-voltage curves of the best performing P3HT:PC_60_BM bulk heterojunction OPV devices, obtained using U1-3 and S1-3 at a P3HT:PC_60_BM blend ratio of 1:0.7 (by mass). The pixel area was 0.06 cm^2^.

Bulk heterojunction (BHJ) solar cells of general composition ITO/PEDOT:PSS/P3HT:PC_60_BM/Ca/Al were fabricated from each of the six P3HT samples, using a 1:0.7 blend (by mass) of the relevant sample with PC_60_BM (see SI, Section C for methods). All blend solutions were prepared without filtering. Each substrate supported five pixels of active area 0.06 cm^2^. Figure 4 shows for each sample the current density-voltage (*J-V*) curve of the best performing pixel, measured under 100 mWcm^−2^ AM1.5 solar irradiation. The corresponding average device characteristics are recorded in Table 2, along with the highest efficiency obtained for each set of five pixels (in the case of S2 two pixels were excluded as they were shorted).

U1 and S1 did not fully dissolve, and films formed from these samples showed visible precipitates. Despite this, the U1 devices exhibited a moderate average power conversion efficiency (PCE) of 2.3%. The S1 devices had a substantially lower average PCE of 0.3%, which is likely to be due to the previously mentioned change in molecular weight distribution during removal of the scavenging agent.

After purifying the polymer with acetone (U2 and S2), the short-circuit current density and fill factor increased markedly, resulting in average PCEs of 4.3 and 4.5% respectively. After the final chloroform extraction, the average PCE of the U3 devices increased to 4.8%, while (at 4.2%) the average PCE of the S3 devices remained similar to that of the U2 and S2 devices, which is likely due to inadvertent contamination of the sample by the silica agent.

The improvement in efficiency after the material had been extracted in chloroform, was predominantly due to an increase in the short-circuit current density, with the fill-factor and open-circuit voltage remaining largely unchanged. This suggests that the magnesium salts mainly affect the charge generation efficiency, without substantially altering charge transport and (non-geminate) recombination in the devices. While further investigation is needed to determine the fundamental mechanisms by which the various impurities affect device operation, their complete removal is evidently essential to achieve the highest possible device efficiency.

### P3HT:IC_60_BA Device Performance

**Figure 5:**
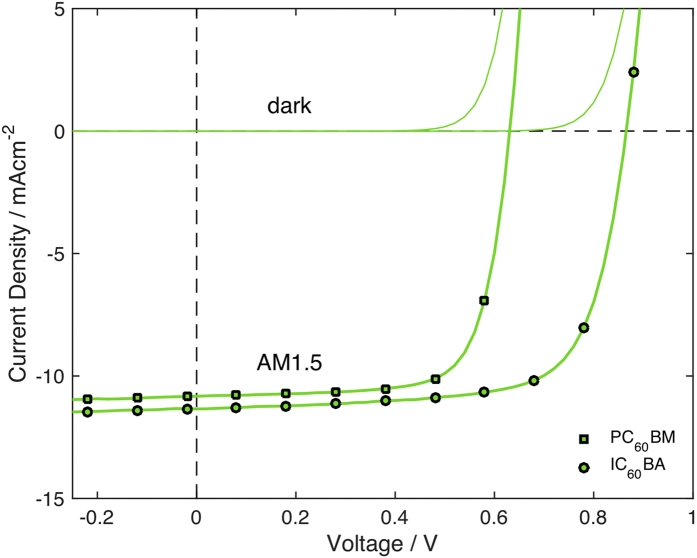
Current density-voltage curves in the dark and under AM1.5 illumination for the best performing P3HT:IC_60_BA bulk heterojunction organic photovoltaic device obtained using sample U3 at a P3HT:IC_60_BA blend ratio of 1:0.7 (by mass). Also shown for comparison is the best performing P3HT:PC_60_BM obtained using sample U3 at the same polymer to fullerene mass ratio.

The best performing material, U3, was tested with an alternative fullerene acceptor indene-C_60_ bis-adduct (IC_60_BA), which has a higher-lying LUMO level of −3.74 eV (compared to −3.91 eV for PC_60_BM), and therefore results in higher open-circuit voltages in BHJ solar cells^26^,^27^,^28^. Using IC_60_BA, polymer U3, and the same device architecture as for the PC_60_BM devices, we recorded average power conversion efficiencies of 6.6% (7.0% peak), confirming the high performance of the fully purified U3 sample. Figure 5 shows current density-voltage curves for the best performing IC_60_BA device in the dark and under illumination, together with comparative data for the best performing PC_60_BM device.

The increase in efficiency relative to PC_60_BM is predominantly due to a substantial increase in average open-circuit voltage from 0.63 to 0.86 V, along with a slight increase in average short-circuit current density from 10.7 to 10.9 mAcm^−2^. The average device characteristics are recorded in Table 3, along with the peak power conversion efficiency for each set of five pixels. The efficiency obtained with IC_60_BA is towards the upper end of efficiencies reported for a non-tandem P3HT:C_60_-fullerene bulk heterojunction device, and highlights the need for thorough polymer purification in order to realise the full potential of the blend system.

## Conclusion

Using XRF and NMR spectroscopy as non-destructive techniques for probing polymer purity, we have investigated the effect of impurities in P3HT:fullerene photovoltaic devices, where the P3HT was synthesised via Grignard metathesis polymerization. By separately removing the excess monomer and salt by-products, we have shown that the presence of monomer/oligomer impurities has the greatest impact on device performance, causing a substantial lowering of short-circuit current density and fill factor. However, removal of all impurities, including the salt by-products, is required to obtain the highest device efficiencies (mean PCEs of 4.8 and 6.6% with PC_60_BM and IC_60_BA, respectively). The inclusion of nickel-targeting silica-supported metal scavenging agents was found to introduce silica impurities into the sample, which impair device performance. Whilst this study has focused specifically on by-products produced during Grignard metathesis polymerization, by-products produced during Stille, Suzuki and direct arylation polymerizations can be expected to have similar effects on solution processability and device performance. The development of fast, simple and effective purification procedures is therefore likely to be critical to the development of improved materials that are competitive with their conventional inorganic counterparts.

## Additional Information

**How to cite this article**: Bannock, J. H. *et al*. The influence of polymer purification on the efficiency of poly(3-hexylthiophene):fullerene organic solar cells. *Sci. Rep.* **6**, 23651; doi: 10.1038/srep23651 (2016).

## Supplementary Material

Supplementary Information

## Figures and Tables

**Table 1:** Weight-average molecular weight, peak molecular weight and polydispersity of U1-3 and S1-3, determined by size-exclusion chromatography (see SI, Sections C and F).

Sample	Mw/kgmol^−1^	Mp/kgmol^−1^	PDI
U1	124	125	1.8
S1	76	58	2.5
U2	122	124	1.9
S2	127	125	1.8
U3	121	117	1.8
S3	127	126	2.0

**Table 2:** Average device parameters for 0.06 cm^2^ P3HT:PC_60_BM bulk heterojunction OPV devices fabricated using samples U1-3 and S1-3 at a P3HT:PC_60_BM blend ratio of 1:0.7 (by mass); *V*_oc_ = average open-circuit voltage; *J*_sc_ = average short-circuit current density; *FF* = average fill factor; *η*_av_ = average power conversion efficiency; and *η*_peak_ = peak power conversion efficiency for the best-performing cell.

Sample	V_oc_/V	J_sc_/mAcm^−2^	FF	η_av_/%	η_peak_/%
U1	0.63 ± 0.005	5.9 ± 0.18	62.0 ± 0.9	2.3 ± 0.1	2.4
S1	0.55 ± 0.138	1.8 ± 0.04	29.9 ± 1.5	0.3 ± 0.1	0.4
U2	0.64 ± 0.002	9.6 ± 0.29	69.6 ± 1.0	4.3 ± 0.2	4.5
S2*	0.63 ± 0.003	9.9 ± 0.18	71.7 ± 0.6	4.5 ± 0.1	4.6
U3	0.63 ± 0.001	10.7 ± 0.14	70.9 ± 1.6	4.8 ± 0.2	5.0
S3	0.62 ± 0.003	9.6 ± 0.13	70.5 ± 0.7	4.2 ± 0.1	4.4

Average values were obtained using data from five cells; quoted error bounds correspond to one standard deviation. (*Two pixels were excluded due to shorting).

**Table 3:** Average device parameters for 0.06 cm^2^ P3HT:IC_60_BA bulk heterojunction organic photovoltaic devices fabricated using sample U3 at a P3HT:IC_60_BA blend ratio of 1:0.7 (by mass).

Fullerene	V_oc_/V	J_sc_/mAcm^−2^	FF	η_av_/%	η_peak_%
PC_60_BM	0.63 ± 0.001	10.7 ± 0.14	70.9 ± 1.6	4.8 ± 0.2	5.0
IC_60_BA	0.86 ± 0.006	10.9 ± 0.56	70.0 ± 1.7	6.6 ± 0.5	7.0

*V*_oc_ = average open-circuit voltage; *J*_sc_ = average short-circuit current density; *FF* = average fill factor; *η*_av_ = average power conversion efficiency; and *η*_peak_ = peak power conversion efficiency for the best-performing cell. Average values were obtained using data from five cells; quoted error bounds correspond to one standard deviation.
