# Supplementary material for: The influence of polymer purification on the efficiency of poly(3-hexylthiophene):fullerene organic solar cells
Source: Sci Rep. 2016 Mar 31;6:23651. doi: 10.1038/srep23651 (PMC4814819; doi:10.1038/srep23651)
Supplement: Supplementary Information [file srep23651-s1.docx]

SUPPLEMENTARY INFORMATION

**The influence of polymer purification on the efficiency of**

**poly(3-hexylthiophene):fullerene organic solar cells**

James H. Bannock,^1,2†^ Neil D. Treat,^1,3,4†^ Michael Chabinyc,^4^ Natalie Stingelin,^1,3^

Martin Heeney,^1,2^ and John C. de Mello^1,2*^

* j.demello@imperial.ac.uk

† these authors contributed equally to the work

^1.^Centre for Plastic Electronics, ^2.^Department of Chemistry, ^3.^Department of Materials, Imperial College London, London SW7 2AZ, UK and ^4.^Materials Research Laboratory, University of California Santa Barbara, Santa Barbara, California 93117, USA.

**A. Synthesis of Poly(3-hexythiophene)**

2,5-dibromo-3-hexylthiophene (4 g, 12.3 mmol) (Lanzhou Galaxy) was degassed in a 100 mL round-bottomed flask, and then placed under an argon atmosphere. 40 mL of anhydrous THF (Aldrich) was added to the flask, followed by the dropwise addition of 6 mL of 2M isopropylmagnesium chloride in THF (12 mmol, 0.98 equiv.) (Aldrich). The solution was heated at 55 °C for 30 minutes in an oil bath, resulting in a colour change from pale brown (after addition of the Grignard) to bright yellow. Nickel(II)[1,3-bis(diphenylphosphino)propane] chloride (10 mg, 0.15 mol%) (Aldrich) was added to the solution in a single shot under a positive flow of argon. The reaction solution immediately turned red, indicative of polymerization. The polymerization was quenched after 150 minutes by a single injection of 20 mL of methanol. The flask was removed from the heat and allowed to cool to room temperature. The (gelled) polymer was removed from the flask into a fluted cellulose filter without further addition of methanol, and then allowed to dry.

**B. Expected Yield**

Ignoring bromine chain-ends, the theoretical maximum yield from the reaction is 1.63 g. In practice it is typical to recover in excess of 1.5 g of dry material after sequential Soxhlet extraction of the polymer with acetone followed by chloroform. The slight discrepancy between expected yield and recovered product can be attributed to material loss during work-up.

| **Molecule** | **molar equiv.**  **(to P3HT)** | **FW / gmol^-1^** | **Mass (per gram P3HT) / g** | **% by mass** |
| --- | --- | --- | --- | --- |
| P3HT (**5**) | 1 (0.006^[[1]](#footnote-1)^) | 166.3 | 1 | 30.5 |
| 2-bromopropane | 1.25 | 123.0 | 0.92 | 28.0 |
| MgBrCl | ≅1 | 139.7 | 0.84 | 25.6 |
| MgOMeCl | ≅ 0.25 | 90.8 | 0.14 | 4.3 |
| **4a** | - | - | - | - |
| **4b** | 0.25 | 247.2 | 0.37 | 11.3 |
| Ni(II) salt | 0.0015 | unknown | < 0.01^[[2]](#footnote-2)^ | 0.3^b^ |
|  |  | **Total** | **3.28** |  |

**Table SI1:** Breakdown of reaction by-products formed during GRIM polymerisation of P3HT; values quoted per gram of P3HT.

**C. Experimental Methods**

*Soxhlet Extraction:* Soxhlet extraction was performed using cellulose Soxhlet thimbles (VWR). Soxhlet extraction in acetone was performed for 24 hours. The polymer was then removed from the thimble and allowed to dry in a vacuum oven overnight. The dried polymer was then divided in half. One half of the material was returned to the Soxhlet thimble for chloroform extraction, and the other half of the polymer was kept for samples U2/S2. The polymer in the thimble was then extracted with chloroform until the colour of the solvent in the extractor became colourless (typically 1-3 hours at this scale). (Note: the glass extractor was changed after acetone purification and prior to extraction in chloroform to avoid the risk of cross-contamination).

*Post Extraction Work-Up:* cold methanol was added to the hot chloroform solution containing the polymer, resulting in immediate precipitation of the polymer. The chloroform was then selectively removed on a rotary evaporator (resulting in a slurry solution of the polymer), following which the methanol was removed by continued rotary evaporation. The powder was allowed to dry in a vacuum oven overnight, resulting in a fine dark powder with a green/gold iridescent colour.

*Gel Permeation Chromatography / Size Exclusion Chromatography (GPC-SEC):* polymers were analysed using an Agilent 1200 series GPC-SEC instrument, running filtered chlorobenzene at 1 mL/min. The instrument was fitted with two PLgel mixed-B columns and a single mixed-B guard column. Columns were maintained at a constant temperature of 80 °C. Eluting polymer chains were detected using a refractive index (RI) detector, and molecular weight distributions were determined relative to polystyrene standards.

*Absorption Spectroscopy:* samples were dissolved in chlorobenzene and analysed in 10 mm quartz cuvettes using a Jobin-Yvon Fluoromax-2 fluorimeter fitted with a transmission photodiode.

*NMR spectroscopy:* samples were analysed at room temperature on Bruker 400 MHz instruments. Samples were prepared in deuterated chloroform (Merck) and gently heated to aid dissolution.

*X-ray Fluorescence Spectroscopy:* spectra were obtained from powder samples of each polymer using a Bruker AXS S4 Explorer spectrometer and analysed using the ’Bruker SPECTRA plus’ software package.

*P3HT:PC_60_BM OPV devices:* solar cells of general composition ITO/PEDOT:PSS/P3HT:PC_60_BM/ Ca/Al were fabricated as follows: poly(3,4-ethylenedioxythiophene)-poly(styrenesulfonate) PEDOT: PSS (Clevios PVP AI 4083) was spin-coated at 4000 rpm onto indium tin oxide coated glass and annealed at 175 °C to form a 35-40 nm thin film. For each polymer, a 30 mg/mL solution of P3HT in chlorobenzene was mixed with a 45 mg/mL stock solution of PC_60_BM (Solenne, used as supplied) in chlorobenzene to form a 1:0.7 (by mass) blend of P3HT:PC_60_BM. The polymer:fullerene solutions were spin-coated at 1000 rpm without prior filtration to form a 70-80 nm thin film. The resulting films were annealed at 160 °C for 10 minutes. Cathodes consisting of 15 nm calcium capped with 80 nm aluminium were evaporated onto the active layer, forming five 0.06 cm^2^ cells on a single substrate.

*P3HT:indene-C_60_ bis-adduct (IC_60_BA) OPV devices:* were prepared in the same way using a 1:0.7 blend (by mass) of polymer to fullerene, except a 1.5 vol% loading of chloronapthalene was added to the blend solution to aid phase segregation. IC_60_BA was obtained from Solenne and used as supplied.

*OPV Device Characterisation:* all devices were tested using a Keithley 2408 sourcemeter under 100 mWcm^-2^ simulated AM1.5G solar irradiation (Newport) without the use of an aperture. The illumination intensity was measured using a standard silicon diode, as previously reported.^1,2^

**D. Scavenger efficiency**

The effect of ethylenediamine-modified silica scavenging agents (BASF MSA-FC Si-1, 50-83 μm) on Ni(dppp)Cl­_2_ was investigated by making a 1 mg/mL solution of Ni(dppp)Cl­_2_ in anhydrous THF, along with a 1:1 loading (by mass) of the dppp ligand, see inset photograph in Figure SI1 (left). The addition of the dppp ligand is essential for preventing dissociation of the dppp from the nickel, which would otherwise render the nickel catalytically inactive.^3^ A portion of the stock solution was added to a second vial containing the scavenging agent. The solution was stirred at room temperature for 5 minutes, resulting in the loss of the orange/red colour, see inset photograph in Figure SI1 (right). At the same time the scavenging agent turned from white to pale green.

The solution was filtered with a 0.45 μm PTFE filter to remove the scavenging agent. To confirm the loss of nickel from the solution, the absorption spectra of the solution before and after adding the agent were recorded along with absorption spectrum of the ligand (see Figure SI1). After addition of the agent, the absorption spectrum of the scavenged solution resembled that of a pure dppp solution in THF, with no other features that might indicate the presence of residual nickel species in solution.

**Figure SI1:** Absorption spectra of Ni(dppp)Cl_2_/dppp in THF before adding the metal scavenger (blue line); and after adding the scavenging agent (red line); also shown for comparison is the absorption spectrum of the dppp ligand in THF (black dashed line). Inset photograph: Ni(dppp)Cl_2_/dppp in THF before (left) and after (right) adding the metal scavenger. The scavenging agent at the bottom of the vial assumed a pale green colour after coordinating to the nickel.

**E. Application of Metal Scavenging Agent to Polymer Samples**

An aliquot of the relevant polymer was dissolved in hot THF (and chloroform for S1), and an empirical measure of the ethylenediamine-modified silica scavenging agent was added (greater than the quantity needed to trap all of the catalyst). The solution was stirred for 15 min and then hot-filtered (cellulose, Whatman, Grade 3, pore size: 6 μm *cf.* 50-83 μm for the silica agent) under vacuum to remove the scavenging agent from the solution.

**F. GPC-SEC chromatograms**

**Figure SI2:** Elugrams of samples U1-3 and S1-3, as determined by size-exclusion chromatography using refractive index detection.

Molecular weight distributions for the six samples were extracted from the raw elugrams (Figure SI2) by converting elution time to molar mass using a calibration against well-defined polystyrene standards and approximating the logarithm of the molar mass as a linear function of the elution time. The corresponding molecular weight distributions are shown in Figure SI3.

**Figure SI3:** Molecular weight distributions of samples U1-3 and S1-3, extracted from the data shown in Fig. SI2.

**G. Normalised Absorption Spectra**

**
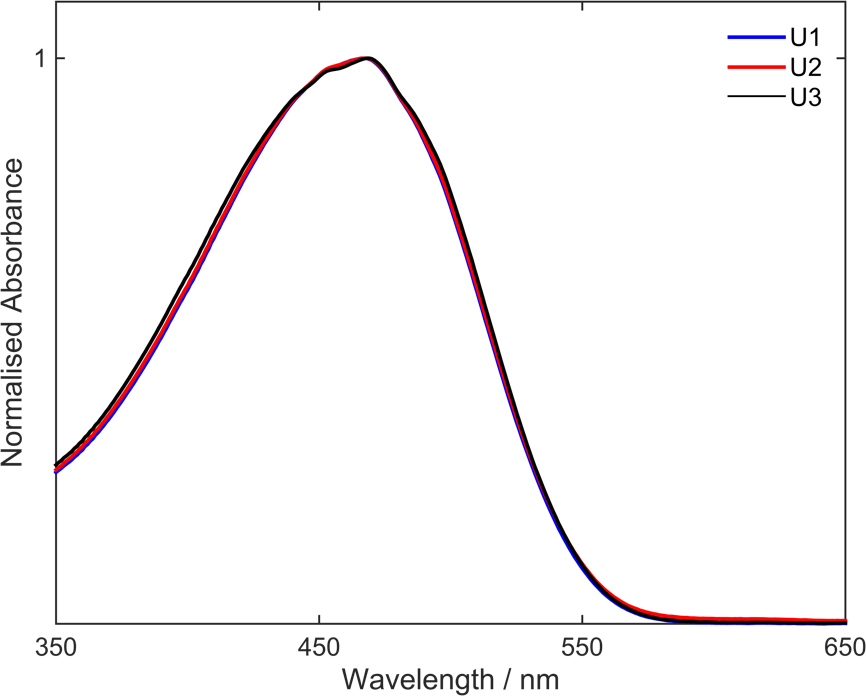
**

**Figure SI4:** Normalised absorption spectra of U1-3 (recorded in chlorobenzene).

**H. Full range ^1^H NMR spectra**

**Figure SI5:** Full range ^1^H NMR spectra of samples U1-U3. Note, the slight signal at 4.0 ppm in the case of U2 is due to residual IPA solvent on the NMR tube.

**I. X-Ray Fluorescence Spectrometry**

Spectra are provided at the end of this section.

| **Element**  **/ E** | **U1** | | **S1** | | **U2** | | **S2** | | **U3** | | **S3** | |
| --- | --- | --- | --- | --- | --- | --- | --- | --- | --- | --- | --- | --- |
|  | **[E]** | **[E_R_]** | **[E]** | **[E_R_]** | **[E]** | **[E_R_]** | **[E]** | **[E_R_]** | **[E]** | **[E_R_]** | **[E]** | **[E_R_]** |
| **S** | 31.55 | 1 | 43.94 | 1 | 53.07 | 1 | 74.53 | 1 | 98.18 | 1 | 92.26 | 1 |
| **Br** | 47.47 | 1.5 | 39.43 | 0.9 | 46.81 | 0.88 | 0.758 | 0.01 | 0.59 | <0.01 | 0.355 | <0.01 |
| **Cl** | 11.6 | 0.37 | 7.84 | 0.18 | 8.14 | 0.15 | 3.3 | 0.04 | - |  | - |  |
| **Mg** | 7.99 | 0.25 | 5.53 | 0.13 | 15.0 | 0.28 | 5.9 | 0.08 | - |  | - |  |
| **Si** | 1.2 | 0.04 | 2.79 | 0.06 | - | - | 14.9 | 0.20 | 0.52 | <0.01 | 6.76 | 0.07 |
| **Ni** | 0.082 | <0.01 | 0.085 | <0.01 | 0.21 | <0.01 | 0.066 | <0.01 | - | - | - | - |

**Table SI2:** Elemental concentrations for samples U1-3 and S1-3 determined by XRF. [E] denotes the percentage by mass of element E in each sample, [E_R_] denotes the mass ratio of element E relative to sulfur.

The molar concentration of each element E was calculated by dividing the mass concentration [E] by the atomic mass of element E. The molar concentration of each element was then normalised to the molar concentration of sulfur to obtain the molar equivalence relative to sulfur.

To enable the polymer purity to be calculated, the mass of sulfur was scaled-up to the mass of the polymer repeat unit (FW(C_10_H_14_S)/A_r_(S) = 166.3/32.1 = 5.18) to take into account the mass of the undetected carbon and hydrogen atoms in the polymer structure. The percentage purity of the polymer was then determined from the percentage mass of the polymer relative to the total mass of all components in the sample, as defined in Equation (S1) (where *m_i_* is the mass of element/molecule *i*).

$\% \text{purity}=100\% \times\frac{m_{\text{polymer}}}{\sum_{i} m_{i}}$ (S1)

Note, trace quantities of Ca, Fe, Pd, Re and Zn were excluded from the analysis since the occurrence of these elements in the samples was assumed to arise from standard laboratory utensils.


**Figure SI6:** XRF spectrum of U1.

**Figure SI7:** XRF spectrum of S1.

**Figure SI8:** XRF spectrum of U2.

**Figure SI9:** XRF spectrum of S2.

**Figure SI10:** XRF spectrum of U3.

**Figure SI11:** XRF spectrum of S3.

**J. External Quantum Efficiency**

The external quantum efficiency (EQE) of a fully purified P3HT:IC_60_BA device was measured using a separate batch of P3HT that had been subjected to the same purification protocol as U3, see Fig. SI12. (See Ref. 2 for a description of the experimental set up). The current-voltage response of the same device is provided in Fig. SI13.

**Figure SI12:** External quantum efficiency versus wavelength for a P3HT:IC_60_BA device fabricated using P3HT that had been treated with an acetone wash, followed by a chloroform extraction. (Note, although purified using the same protocol as U3, the sample was prepared from a different batch of polymer). The spectral response was recorded after twenty four hours of storage in a dry N_2_ glovebox.

**Figure SI13:** Current voltage trace for the device from Figure SI12 under simulated AM1.5G illumination, recorded immediately after device fabrication. The short-circuit current, open-circuit voltage and fill-factor were 10.5 mA/cm^2^, 0.86 V and 70 %, respectively, corresponding to an overall PCE of 6.3 %. Note, this value is slightly different from that measured for U3 due to the use of a different polymer batch.

**K. References**

1. Peet, J. *et al.* Efficiency enhancement in low-bandgap polymer solar cells by processing with alkane dithiols. *Nat. Mater.* **6,** 497–500 (2007).

2. Treat, N. D. *et al.* Polymer-fullerene miscibility: A metric for screening new materials for high-performance organic solar cells. *J. Am. Chem. Soc.* **134,** 15869–15879 (2012).

3. Bannock, J. H. *et al.* Controlled synthesis of conjugated random copolymers in a droplet-based microreactor. *Mater. Horiz.* **1,** 214–218 (2014).

1. mol/g P3HT, assuming FW: 166.3 g/mol [↑](#footnote-ref-1)
2. calculation based on Ni(dppp)Cl_2_ [↑](#footnote-ref-2)
